# Supplementary material for: MDM2 inhibitors, nutlin-3a and navtemadelin, retain efficacy in human and mouse cancer cells cultured in hypoxia
Source: Sci Rep. 2023 Mar 20;13:4583. doi: 10.1038/s41598-023-31484-0 (PMC10027891; doi:10.1038/s41598-023-31484-0)
Supplement: Supplementary file 1 — Supplementary Information. [file 41598_2023_31484_MOESM1_ESM.pdf]

SUPPLEMENTARY INFORMATION

MDM2 inhibitors, nutlin-3a and navtemadelin, retain efficacy in human and mouse cancer cells cultured in hypoxia

Ada Lerma Clavero, Paula Lafqvist Boqvist, Katrine Ingelshed, Cecilia Bosdotter,
Saikiran Sedimbi, Long Jiang, Fredrik Wermeling, Borivoj Vojtesek,
David P. Lane, Pavitra Kannan

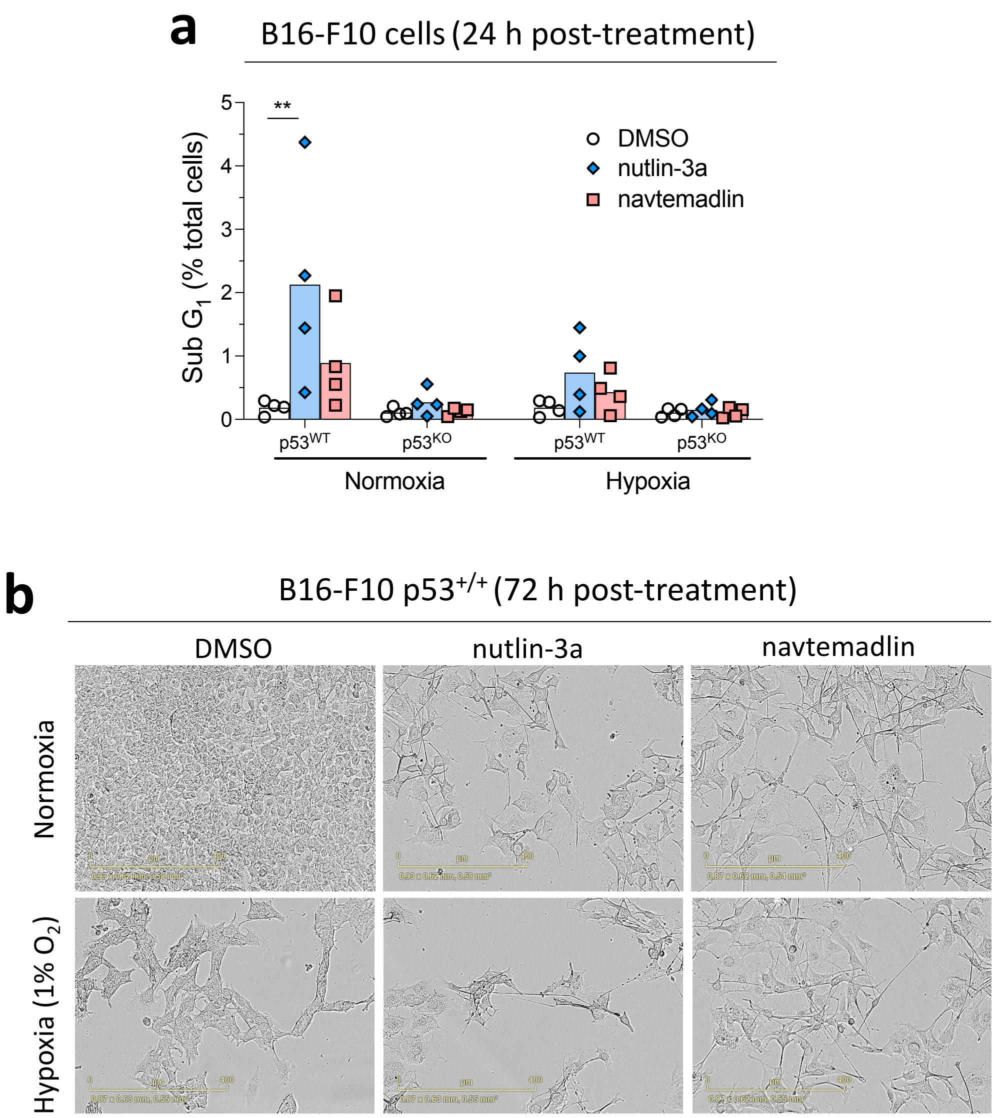

Supplementary Figure 1. *MDM2 inhibitors induce growth arrest and apoptosis in mouse B16-F10 p53$^{+/+}$ cells after 72 h treatment under normoxia and hypoxia.* **(a)** Percentage of sub-G_1 B16-F10 p53$^{+/+}$ and p53$^{-/-}$ cells after 24 h treatment with DMSO, nutlin-3a (10 μM), or navtemadlin (2 μM) in normoxia or hypoxia. Bar graphs represent the mean value from four independent experiments; individual data points represent mean value from one experiment. Statistical significance was assessed by 2-way ANOVA on log-transformed data followed by post-hoc Dunnett's correction for multiple testing (unpaired, two-tailed, α = 0.05). Adjusted *P*-values from the post-hoc t-tests are indicated (**$P < 0.01$). **(b)** Phase-contrast images of B16-F10 p53$^{+/+}$ cells after 72 h treatment with DMSO, nutlin-3a (10 μM), or navtemadlin (2 μM) in normoxia or hypoxia.

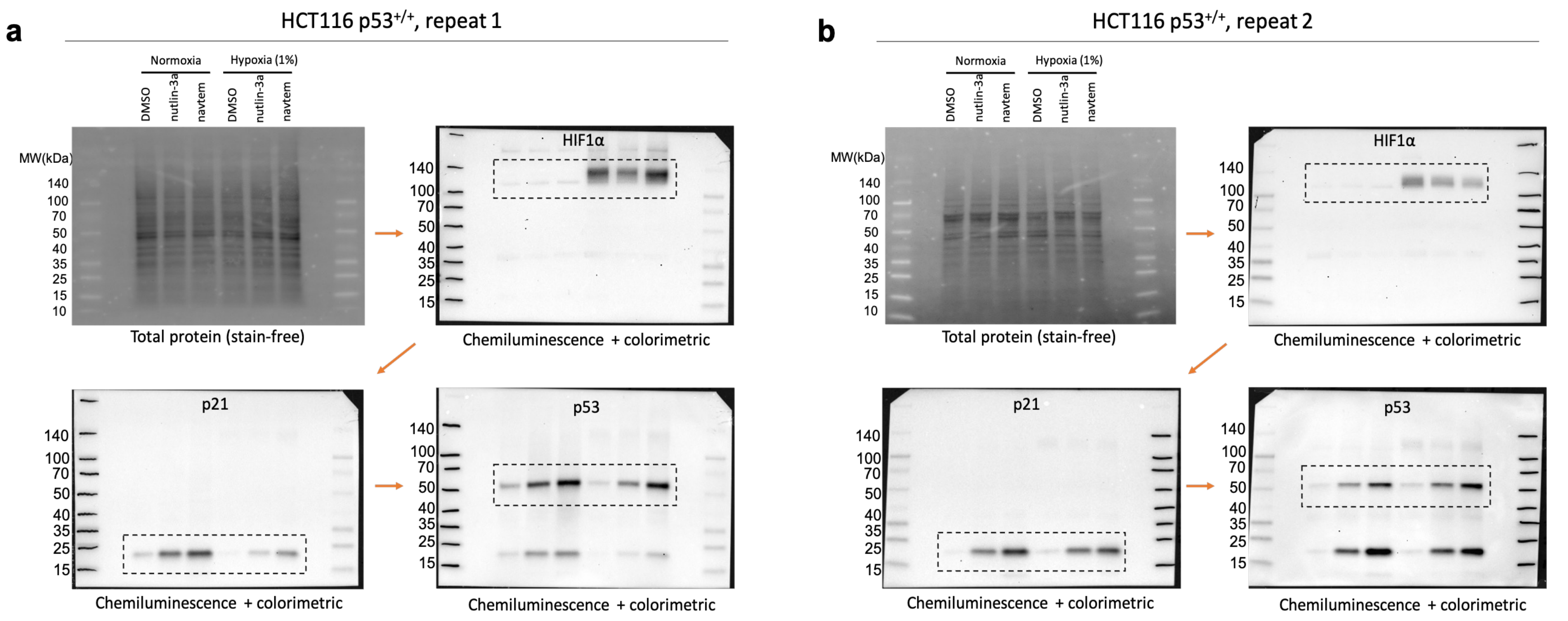

Supplementary Figure 2. *Whole blots from Figure 4 for HCT116 p53$^{+/+}$.* **(a-b)** Blots from repeats 1 and 2 showing total protein (stain-free membrane, top left panel), and target protein (merged chemiluminescence and colorimetric) detected using antibodies against HIF1α (top right panel), p21 (bottom left panel), and p53 (bottom right panel). The whole membrane was incubated with each antibody separately, imaged, and washed prior to addition of the subsequent antibody. Proteins were probed in the order indicated by the arrows.

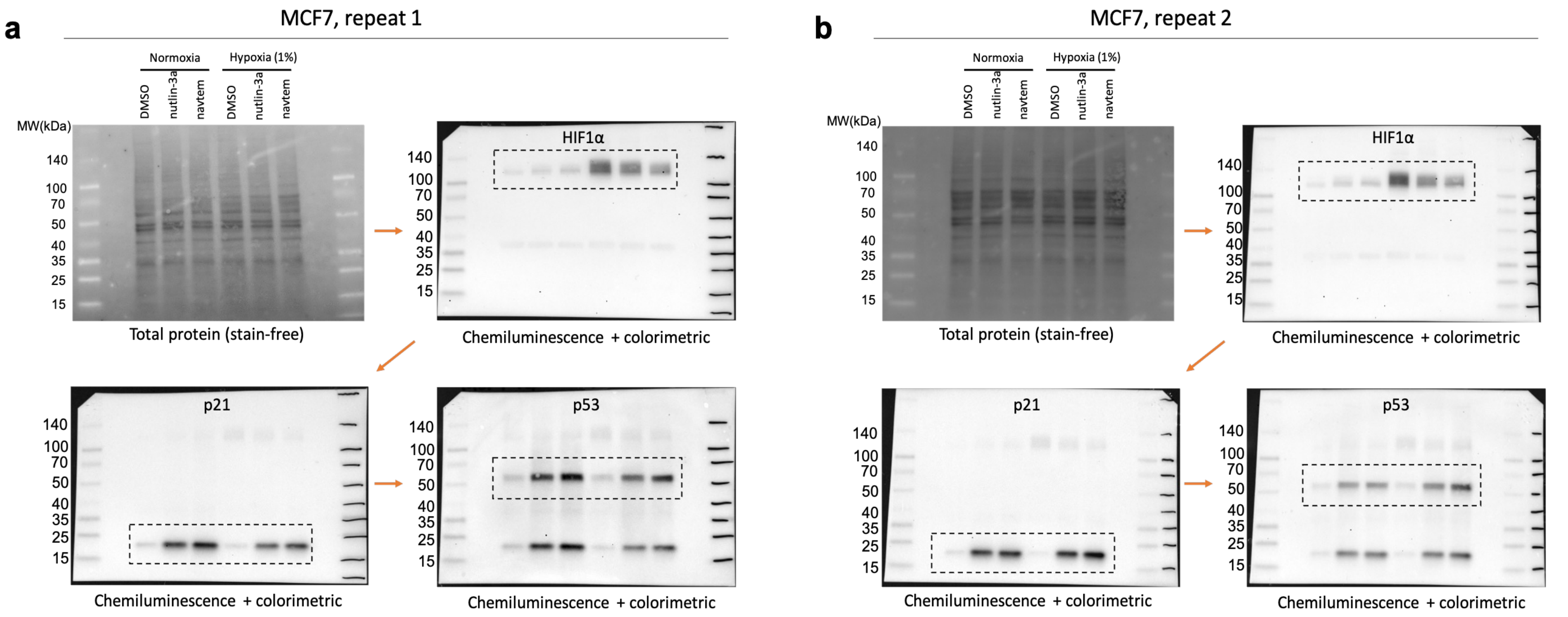

Supplementary Figure 3. *Whole blots from Figure 4 for MCF7.* **(a-b)** Blots from repeats 1 and 2 showing total protein (stain-free membrane, top left panel), and target protein (merged chemiluminescence and colorimetric) detected using antibodies against HIF1α (top right panel), p21 (bottom left panel), and p53 (bottom right panel). The whole membrane was incubated with each antibody separately, imaged, and washed prior to addition of the subsequent antibody. Proteins were probed in the order indicated by the arrows.

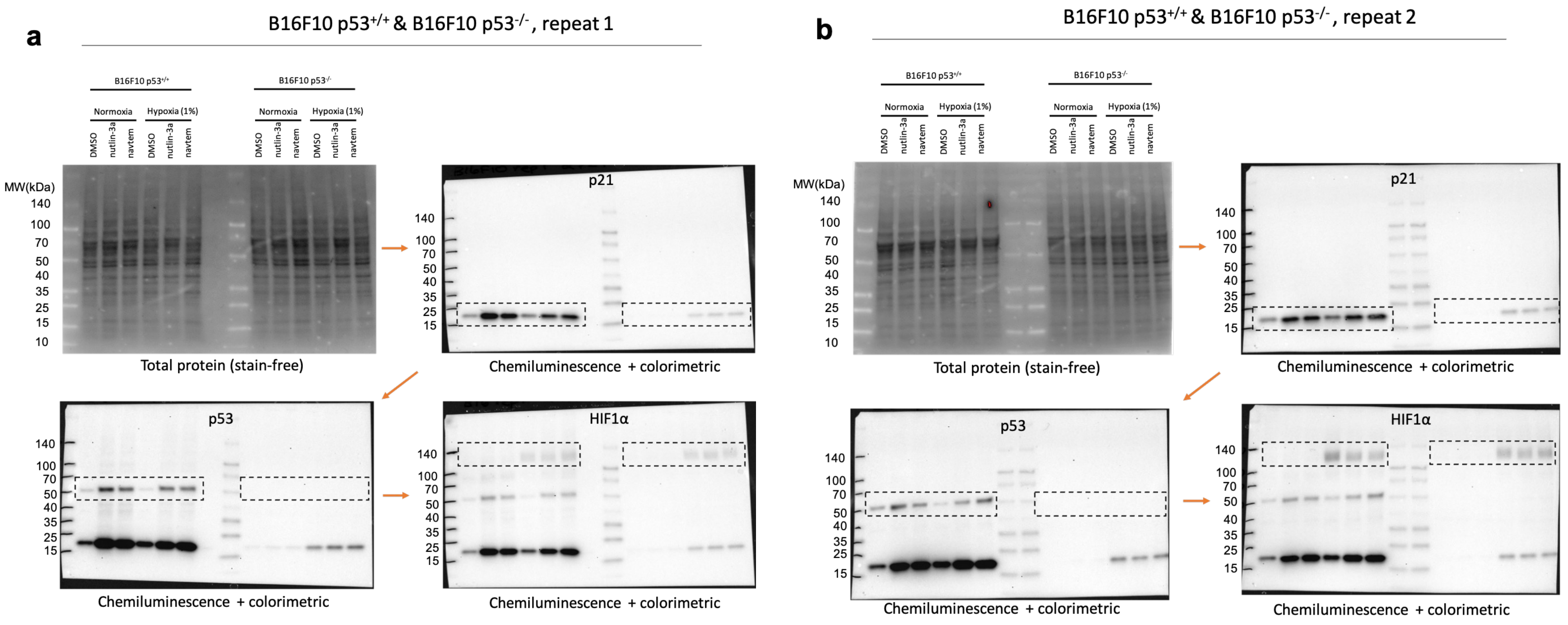

Supplementary Figure 4. *Whole blots from Figure 4 for B16-F10 p53$^{+/+}$ and B16-F10 p53$^{-/-}$.* **(a-b)** Blots from repeats 1 and 2 showing total protein (stain-free membrane, top left panel), and target protein (merged chemiluminescence and colorimetric) detected using antibodies against p21 (top right panel), p53 (bottom left panel), and HIF1α (bottom right panel). The whole membrane was incubated with each antibody separately, imaged, and washed prior to addition of the subsequent antibody. Proteins were probed in the order indicated by the arrows.

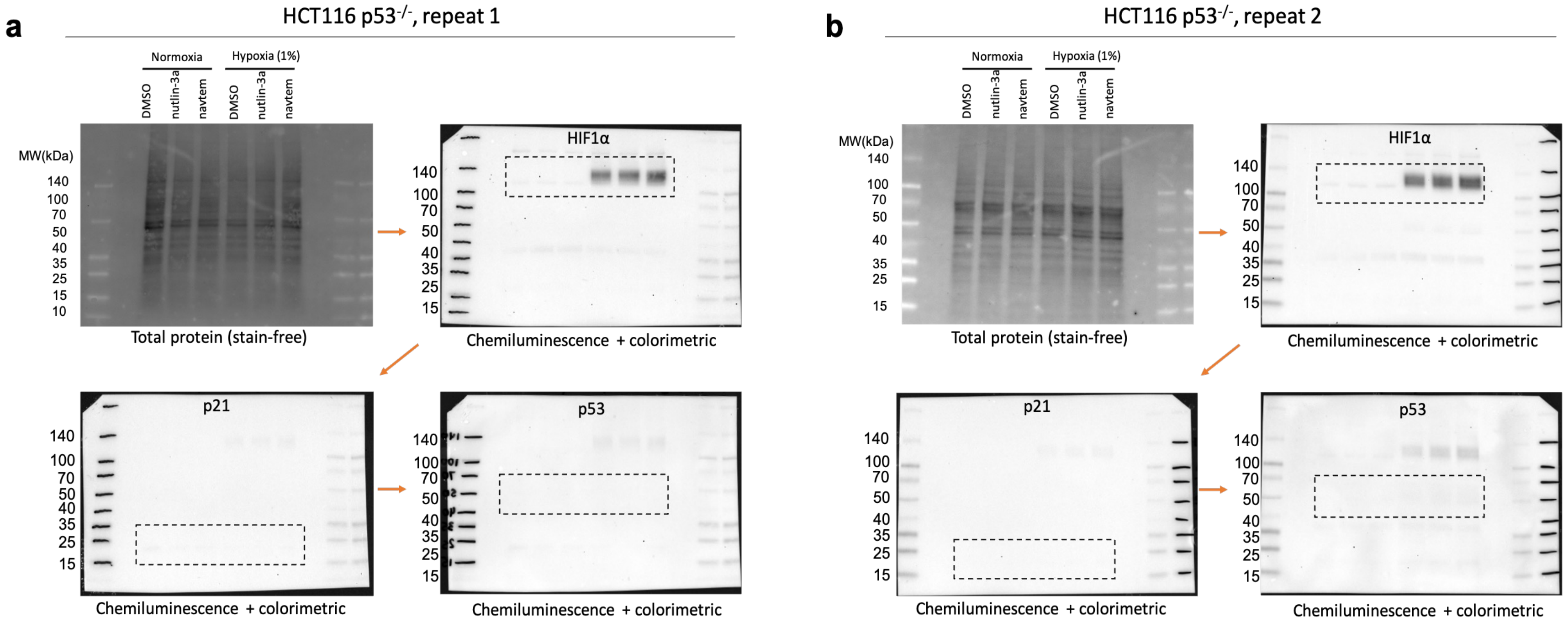

Supplementary Figure 5. *Whole blots from Figure 4 for HCT116 p53$^{-/-}$.* **(a-b)** Blots from repeats 1 and 2 showing total protein (stain-free membrane, top left panel), and target protein (merged chemiluminescence and colorimetric) detected using antibodies against HIF1α (top right panel), p21 (bottom left panel), and p53 (bottom right panel). The whole membrane was incubated with each antibody separately, imaged, and washed prior to addition of the subsequent antibody. Proteins were probed in the order indicated by the arrows.
